# Crucial genes associated with diabetic nephropathy explored by microarray analysis

**DOI:** 10.1186/s12882-016-0343-2

**Published:** 2016-09-09

**Authors:** Zhikui Wang, Zhaoxia Wang, Zhongqi Zhou, Yueqin Ren

**Affiliations:** Department of Nephrology, Linyi People’s Hospital, No.27 Jiefang Road, Lanshan District, Linyi, Shandong 276003 China

**Keywords:** Diabetic nephropathy, Differentially expressed gene, MicroRNA, Transcription factor, Network

## Abstract

**Background:**

This study sought to investigate crucial genes correlated with diabetic nephropathy (DN), and their potential functions, which might contribute to a better understanding of DN pathogenesis.

**Methods:**

The microarray dataset GSE1009 was downloaded from Gene Expression Omnibus, including 3 diabetic glomeruli samples and 3 healthy glomeruli samples. The differentially expressed genes (DEGs) were identified by LIMMA package. Their potential functions were then analyzed by the GO and KEGG pathway enrichment analyses using the DAVID database. Furthermore, miRNAs and transcription factors (TFs) regulating DEGs were predicted by the GeneCoDis tool, and miRNA-DEG-TF regulatory network was visualized by Cytoscape. Additionally, the expression of DEGs was validated using another microarray dataset GSE30528.

**Results:**

Totally, 14 up-regulated DEGs and 430 down-regulated ones were identified. Some DEGs (e.g. *MTSS1*, *CALD1* and *ACTN4*) were markedly relative to cytoskeleton organization. Besides, some other ones were correlated with arrhythmogenic right ventricular cardiomyopathy (e.g. *ACTN4*, *CTNNA1* and *ITGB5*), as well as complement and coagulation cascades (e.g. *C1R* and *C1S*). Furthermore, a series of miRNAs and TFs modulating DEGs were identified. The transcription factor LEF1 regulated the majority of DEGs, such as *ITGB5*, *CALD1* and *C1S*. Hsa-miR-33a modulated 28 genes, such as *C1S*. Additionally, 143 DEGs (one upregulated gene and 142 downregulated genes) were also differentially expressed in another dataset GSE30528.

**Conclusions:**

The genes involved in cytoskeleton organization, cardiomyopathy, as well as complement and coagulation cascades may be closely implicated in the progression of DN, via the regulation of miRNAs and TFs.

**Electronic supplementary material:**

The online version of this article (doi:10.1186/s12882-016-0343-2) contains supplementary material, which is available to authorized users.

## Background

Diabetic nephropathy (DN) is a complication correlated with both type 1 and type 2 diabetes and is characterized by glomerulosclerosis due to accumulation of extracellular matrix [1]. Despite great attention from both clinicians and basic scientists, the morbidity of end-stage renal disease and glomerulosclerosis in diabetic patients is increasing dramatically [2].

In the past years, many molecules associated with DN have been uncovered. For example, the mammalian target of rapamycin (mTOR) complex 1 activation has a key role in podocyte dysfunction in diabetic mice [3, 4]. Rooney et al. have shown that connective tissue growth factor/CCN family protein 2 (CTGF/CCN2) can activate the Wnt signaling in mesangial cells through low density lipoprotein receptor-related protein 6 (LRP6), which may be implicated in the pathogenesis of DN [5]. In diabetic mice, *Glo1* (Glyoxalase 1) overexpression completely inhibits diabetes-induced increases in methylglyoxal modification of glomerular proteins, and promotes the development of diabetic kidney disease (DKD) [6]. Furthermore, there is evidence that miR-192 enhances collagen expression by regulating the E-box repressors Zeb1/2, and locked nucleic acid–anti-miR-192 alleviates proteinuria in the diabetic mice [7]. Based on a gene expression profiling of DN, Hans et al. have shown that some genes in glomeruli from patients with DN are down-regulated, such as bone morphogenetic protein 2, fibroblast growth factor 1, vascular endothelial growth factor, nephrin and insulin-like growth factor binding protein 2, suggesting that progression of DN might be due to diminished tissue repair ability [8]. However, the pathways which the differentially expressed genes (DEGs) participate in and regulators that target these genes remain unknown.

In this study, the microarray dataset GSE1009 deposited by Hans [8] was used to identify DEGs between diabetic glomeruli samples and healthy controls. Gene Ontology (GO) and pathway enrichment analyses were then performed for the up- and down-regulated DEGs. Furthermore, microRNAs (miRNAs) and transcription factors (TFs) regulating DEGs were predicted, and miRNA-DEG-TF regulatory network was constructed. These findings may contribute to a better understanding of the nosogenesis of DN.

## Methods

### Affymetrix microarray data

The gene expression profile data of GSE1009 [8] were downloaded from the public database Gene Expression Omnibus (GEO, http://www.ncbi.nlm.nih.gov/geo/), which was based on the platform of [HG_U95Av2] Affymetrix Human Genome U95 Version 2 Array (GPL8300, Affymetrix Inc., Santa Clara, California, USA). This dataset contained 6 glomeruli samples, including 3 samples from 2 kidneys from patients with diabetes mellitus type 2, and 3 samples from 2 healthy kidneys.

Another gene expression dataset GSE30528 [9] in GEO, which contains a relatively big sample size and has a high data quality, was used for validation. The data in GSE30528 were produced by the platform of [HG-U133A_2] Affymetrix Human Genome U133A 2.0 Array (GPL571, Affymetrix Inc., Santa Clara, California, USA). A total of 9 diabetic glomeruli samples from patients with DKD and 13 healthy glomeruli samples were included in this dataset.

CEL files and the probe annotation files were downloaded, and the gene expression data of all samples were preprocessed via background correction, quantile normalization and probe summarization using the Robust Multi-array Average (RMA) algorithm in Affy software package of Bioconductor (available at http://www.bioconductor.org/packages/release/bioc/html/affy.html) [10].

### DEGs screening

Linear Models for Microarray Data (LIMMA) package [11] of Bioconductor (available at http://www.bioconductor.org/packages/release/bioc/html/limma.html) was used to identify genes that were differentially expressed in diabetic glomeruli. Only the genes meeting *p*-value < 0.05 and |log_2_FC (fold change)| ≥ 1 were chosen as DEGs.

### Enrichment analysis for DEGs

To explore the functions of DEGs in diabetic glomeruli samples, the DAVID (Database for Annotation, Visualization and Integrated Discovery) database [12] was used to perform GO and KEGG (Kyoto Encyclopedia of Genes and Genomes) pathway enrichment analyses for DEGs. The *p*-value < 0.01 and gene count ≥ 2 were set as the cut-off criteria.

Furthermore, the category of enriched GO terms and the gene number were displayed as a histogram which was constructed by WEGO (Web Gene Ontology Annotation Plot) [13] with the cut-off criterion of level = 2.

### Construction of miRNA-DEG-TF regulatory network

GeneCoDis (Gene Annotation Co-occurrence Discovery, http://genecodis.cnb.csic.es/) [14], which is a grid-based tool that integrates biological information from different sources to search for biological characteristics (annotations) that frequently co-occur in a series of genes and rank them by statistical significance, was used to identify miRNAs and TFs regulating DEGs with hypergeometric algorithm, and the adjusted *p*-value < 0.01 was used as the cut-off criteria. The regulatory network consisting of DEGs, miRNAs and TFs were then visualized by Cytoscape (http://cytoscape.org/) [15].

### Validation of the expression level of DEGs

The DEGs between DN and control samples in the dataset GSE30528 were identified using the aforementioned methods. The overlapped DEGs in both of GSE30528 and GSE1009 were then identified using the VennDiagram package. Here, if a gene was differentially expressed in both of GSE30528 and GSE1009 with the same expression pattern (up-regulated or down-regulated expression), this gene was identified as an overlapped DEG.

## Results

### Identification of DEGs

Based on the cut-off criteria, a set of 444 genes were identified to be differentially expressed in the diabetic glomeruli samples, including 14 up-regulated and 430 down-regulated ones, compared with the controls (Additional file 1).

### GO and KEGG pathway enrichment analysis of up- and down-regulated DEGs

**Fig. 1:**
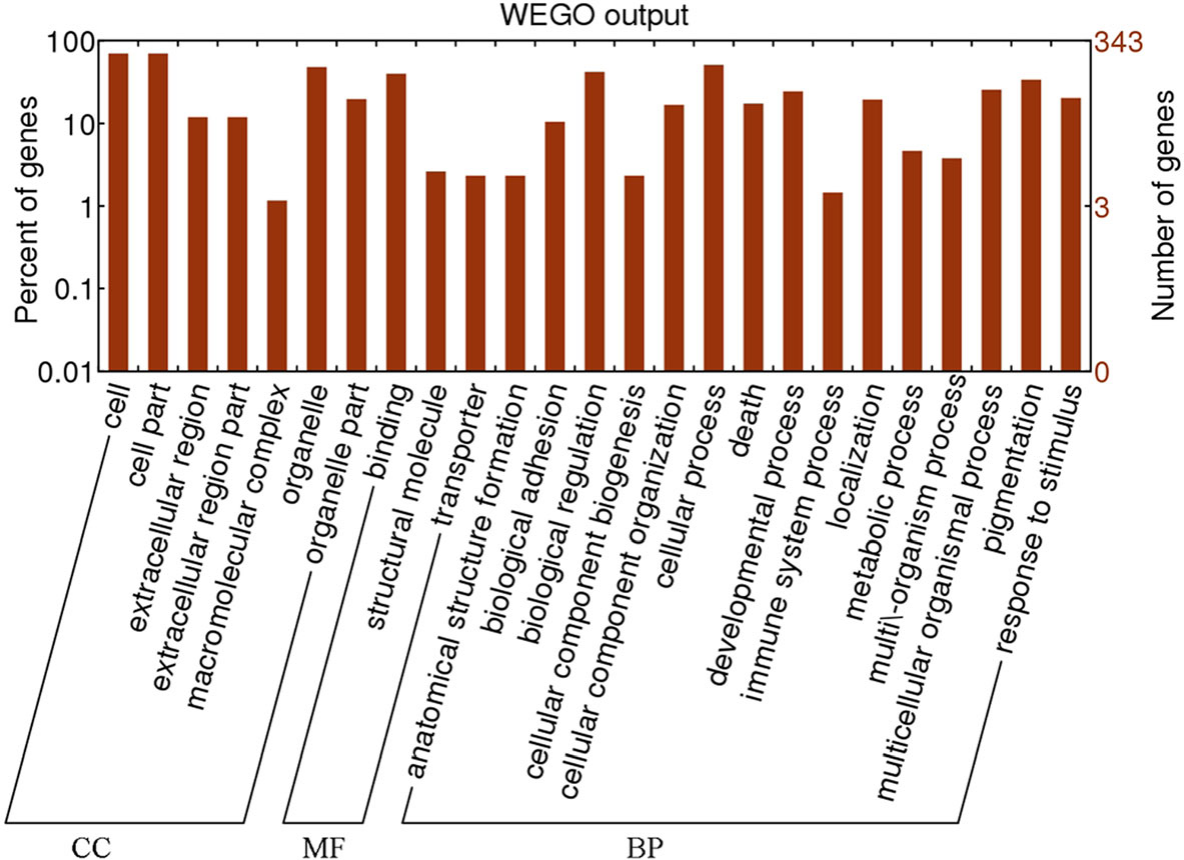
The histogram of the category of enriched GO (Gene Ontology) terms for the down-regulated genes. CC represents cellular component; MF represents molecular function; and BP represents biological process. WEGO represents the tool Web Gene Ontology Annotation Plot

A total of 111 GO terms for the down-regulated DEGs were generated. Some genes (e.g. *MTSS1*, *CALD1* and *ACTN4*) were related to cytoskeleton organization, such as actin filament-based process, actin cytoskeleton and cytoskeletal protein binding; some other genes were distinctly enriched in negative regulation of cell proliferation (e.g. *AIF1*, *IGFBP7* and *COL4A3*) (Table 1). Using WEGO (level = 2), 25 categories of GO terms for down-regulated DEGs were displayed. The majority of genes were enriched in biological processes (BP), such as anatomical structure formation, biological regulation, death and response to stimulus. Moreover, several GO terms in cellular component (CC) were obtained, such as cell part and organelle. Furthermore, three GO terms in molecular function (MF) were enriched by the down-regulated genes, including binding, structural molecule and transporter (Fig. 1). In addition, three up-regulated DEGs (*CD69*, *LGALS2* and *FBP1*) were significantly enriched in sugar binding.

**Table 1:** The top 5 enriched GO terms with the most low *p*-value for differentially expressed genes

Category	Terms	Description	Count	*p*-value	Genes
BP	GO:0030029	actin filament-based process	23	5.38E-07	*MTSS1*, *ROCK1*, *ACTN4*, *AIF1*, *CALD1*, *ARF6*, *MYH9*, *CAPZB*, *TNNT2*, *ACTG1*…
	GO:0030036	actin cytoskeleton organization	22	7.32E-07	*MTSS1*, *ROCK1*, *ACTN4*, *AIF1*, *CALD1*, *ARPC5*, *MYH9*, *CAPZB*, *TNNT2*, *ACTG1*…
	GO:0008285	negative regulation of cell proliferation	28	1.48E-06	*AIF1*, *IGFBP7*, *PTH1R*, *ING1*, *COL4A3*, *BMP2*, *CTBP1*, *GAS1*, *CD164*, *TGFBR3*…
	GO:0007010	cytoskeleton organization	31	2.17E-06	*MTSS1*, *AIF1*, *CALD1*, *ARF6*, *ACTG1*, *PAK1*, *PLS3*, *ACTN4*, *ROCK1*, *MYH9*…
	GO:0030832	regulation of actin filament length	11	6.01E-06	*ACTR3*, *PFN2*, *CAPZA1*, *TMSB4X*, *RDX*, *ARF6*, *ARPC5*, *CAPZB*, *SPTAN1*, *DSTN*…
CC	GO:0015629	actin cytoskeleton	31	2.60E-11	*MTSS1*, *AIF1*, *CALD1*, *MYL9*, *ACTR3*, *ACTG1*, *ACTN4*, *MYO1B*, *MYH9*, *CTNNA1*…
	GO:0005938	cell cortex	20	1.02E-08	*SEPT2*, *ACTN4*, *SEPT1*, *CALD1*, *ARF6*, *MYH9*, *SEPT10*, *ACTG1*, *FNBP1*, *CLIC5*…
	GO:0005856	cytoskeleton	70	1.28E-07	*MTSS1*, *AIF1*, *CALD1*, *MYL9*, *ACTR3*, *ACTG1*, *ACTN4*, *MYO1B*, *MYH9*, *CTNNA1*…
	GO:0044448	cell cortex part	13	1.78E-06	*SEPT2*, *ACTN4*, *SEPT1*, *CALD1*, *PRKCI*, *MYH9*, *CAPZB*, *SEPT10*, *DSTN*, *ACTG1*…
	GO:0043232	intracellular non-membrane-bounded organelle	104	5.54E-06	*SEPT2*, *ACTN4*, *SEPT1*, *CALD1*, *PRKCI*, *MYH9*, *CAPZB*, *SEPT10*, *DSTN*, *ACTG1*…
MF	GO:0008092	cytoskeletal protein binding	40	7.17E-10	*MTSS1*, *AIF1*, *TNNC1*, *CALD1*, *EZR*, *TARDBP*, *ACTN4*, *MYO1B*, *SUN2*, *MYH9*…
	GO:0003779	actin binding	30	5.79E-09	*MTSS1*, *AIF1*, *TNNC1*, *CALD1*, *ACTN4*, *MYH9*, *PALLD*, *NEBL*, *TNNT2*, *PARVA*…
	GO:0005520	insulin-like growth factor binding	6	3.80E-04	*CTGF*, *HTRA1*, *IGFBP7*, *IGFBP2*, *CRIM1*, *CYR61*
	GO:0005200	structural constituent of cytoskeleton	9	6.07E-04	*TNNT2*, *ACTG1*, *DMD*, *VIM*, *AGRN*, *ARPC5*, *CD2AP*, *ADD3*, *SPTAN1*
	GO:0050839	cell adhesion molecule binding	6	9.18E-04	*EZR*, *PVRL2*, *NPTN*, *CTNNA1*, *CD2AP*, *CTNNB1*

*GO* Gene Ontology, *BP* biological process, *MF* molecular function, *CC* cellular component

According to KEGG enrichment analysis, the down-regulated DEGs were significantly enriched in 6 pathways, such as arrhythmogenic right ventricular cardiomyopathy (ARVC) (e.g. *ACTN4*, *CTNNA1* and *ITGB5*), regulation of actin cytoskeleton (e.g. *ACTN4*, *MYL9* and *ITGB5*) and complement and coagulation cascades (e.g. *C1R* and *C1S*). There were no significant pathways for the up-regulated DEGs (Table 2).

**Table 2:** The results of pathway enrichment analysis for the down-regulated genes

Category	Pathway Name	Count	*p*-value	Genes
KEGG_PATHWAY	Arrhythmogenic right ventricular cardiomyopathy (ARVC)	11	1.41E-04	*ACTG1, ATP2A2, ACTN4, DMD, DAG1, CACNB2, ITGB5, ITGA3, CTNNA1, CTNNB1*… *ACTN4, ROCK1, ITGB5, RDX, ITGA3, ARPC5, MYL12A, MYH9, MYL9, ACTG1…*
KEGG_PATHWAY	Regulation of actin cytoskeleton	19	1.65E-04	
KEGG_PATHWAY	Hypertrophic cardiomyopathy (HCM)	9	0.00575	*TNNT2*, *ACTG1*, *ATP2A2*, *TNNC1*, *DMD*, *DAG1*, *CACNB2*, *ITGB5*, *ITGA3*
KEGG_PATHWAY	Complement and coagulation cascades	8	0.00641	*CD55*, *F3*, *CD46*, *CD59*, *C1R*, *SERPING1*, *C1S*, *F2R*
KEGG_PATHWAY	Dilated cardiomyopathy	9	0.00920	*TNNT2*, *ACTG1*, *ATP2A2*, *TNNC1*, *DMD*, *DAG1*, *CACNB2*, *ITGB5*, *ITGA3*
KEGG_PATHWAY	Pathogenic Escherichia coli infection	7	0.00956	*ACTG1*, *EZR*, *ROCK1*, *TUBB2A*, *YWHAQ*, *ARPC5*, *CTNNB1*

KEGG, Kyoto Encyclopedia of Genes and Genomes

### Analysis of miRNA-DEG-TF regulatory network

**Fig. 2:**
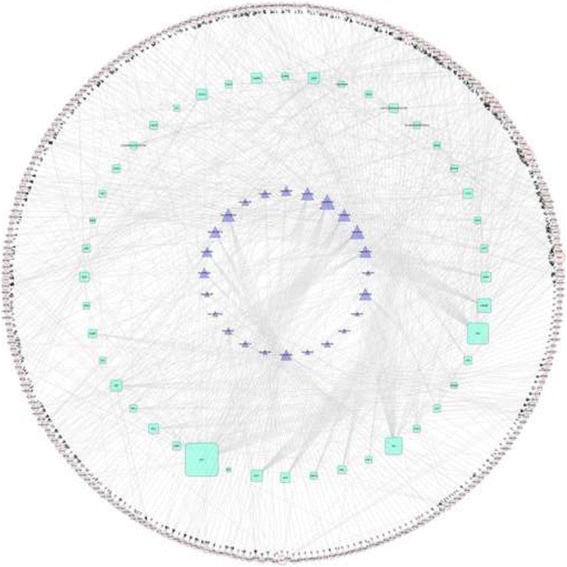
The network consisting of differentially expressed genes, microRNAs and transcription factors. Rounded nodes represent genes; trigonal nodes represent microRNAs; and quadrate nodes represent transcription factors

Totally, 896 regulatory relationships between miRNAs, TFs and DEGs were identified to construct the miRNA-DEG-TF regulatory network, including 24 miRNAs, 44 TFs and 275 down-regulated genes (Fig. 2). The transcription factor *LEF1* regulated most of genes (degree = 79), such as *ITGB5*, *CALD1*, *RTN4* and *PAK1*. Hsa-miR-33a modulated 28 genes, such as *C1S* and *RTN4*.

Besides, no miRNAs were predicted to regulate the up-regulated genes, whereas, two TFs (*AML1* and *NFKB*) were identified to modulate several up-regulated genes. Among them, *AML1* regulated *CD96* and *CYP17A1*.

### Screening of overlapped DEGs in the two datasets

**Fig. 3:**
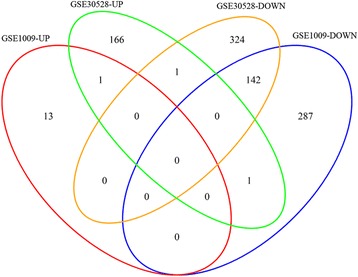
The Venn diagram showing the overlapped differentially expressed genes in the two datasets GSE30528 and GSE1009. “GSE30528-UP” represents the upregulated genes in the dataset GSE30528; “GSE30528-DOWN” represents the downregulated genes in the dataset GSE30528; “GSE1009-UP” represents the upregulated genes in the dataset GSE1009; and “GSE1009-DOWN” represents the downregulated genes in the dataset GSE1009. Arabic numerals in the diagram represent the numbers of the overlapped genes

To validate the expression of the identified DEGs in the dataset GSE1009, another dataset GSE30528 was used. A total of 635 DEGs were identified in GSE30528. Among them, 143 DEGs, including one upregulated gene (*TRIM16*) and 142 downregulated genes (e.g. *MTSS1*, *ACTN4* and *ITGB5*), were also differentially expressed in the dataset GSE1009 (Fig. 3), indicating that the expression of the 143 DEGs in GSE1009 identified above were validated by the dataset GSE30528.

## Discussion

In the present study, a set of 444 DEGs in the dataset GSE1009 were identified from diabetic glomeruli samples, including 14 up-regulated ones and 430 down-regulated ones, compared with healthy glomeruli samples. Among them, the expression of 143 DEGs (one upregulated gene and 142 downregulated genes) were validated by another dataset GSE30528. According to the GO functional enrichment analysis, a set of DEGs were related to the function of cytoskeleton organization, such as *MTSS1*, *ACTN4* and *CALD1*.

*MTSS1* encodes metastasis suppressor 1, which is also known as MIM (missing in metastasis). It is an actin and membrane binding protein that was originally identified as a potential tumor metastasis suppressor [16]. *MTSS1* can induce actin-rich protrusions at the plasmalemma and promote disintegration of actin stress fibers [17], indicating that it may be crucial in regulating cytoskeletal dynamics. Renal tubular cell and podocyte apoptosis is an inevitable event in the progression of glomerulosclerosis [18, 19], and major modifications of the cytoskeleton are involved in the apoptosis progress, including dynamic membrane blebbing, nuclear disintegration, chromatin condensation and cell fragmentation [20]. Therefore, *MTSS1* may play a pivotal role in DN, via participation in the regulation of cytoskeleton organization. *ACTN4* encodes an actinin, which participates in cytoskeleton action. Previous studies have been reported that mutations in *ACTN4* cause focal segmental glomerulosclerosis [21–23]. There is evidence that two single-nucleotide polymorphisms in *ACTN4* are associated with DN in women [24]. Moreover, the up-regulation of *ACTN4* was observed during the proteinuria stage [25]. Thereby, *ACTN4* may be also important in the progression of DN. *CALD1* encodes a caldesmon that plays a key role in the regulation of smooth muscle and nonmuscle contraction [26]. A previous study has shown that *CALD1* is over-expressed in fibroblasts from the diabetic patients with nephropathy, compared with those from the controls [27]. Conway et al. have demonstrated that caldesmon is a candidate susceptibility gene for DN [28]. In this study, *CALD1* was found to be regulated by several TFs, such as *LEF1*. LEF1 (lymphoid enhancer-binding factor 1) is a nuclear transcription factor modulated by Wnt, and it expedites epithelial to mesenchymal transition (EMT) when its activity is activated by β-catenin [29]. Rooney et al. have reported that overexpression of *CCN2* during the progression of DN likely results in the activation of Wnt signaling and subsequent initiation of TCF/LEF transcription [5]. Collectively, *CALD1* may play an essential role in the development of DN likely through the regulation of *LEF1*.

Furthermore, a series of downregulated DEGs were discovered to be enriched in the pathways of arrhythmogenic right ventricular cardiomyopathy and hypertrophic cardiomyopathy in our study, such as *ITGB5*. Diabetic cardiomyopathy is a frequent event in diabetic patients due to hyperglycemia [30]. *ITGB5* encodes integrin beta 5 [31], and it has been found to be differentially expressed in DKD glomeruli and tubuli [9]. Furthermore, integrins are the primary receptors for intercellular adhesion molecule 1 (ICAM1), which has been reported to have a close relationship with DN [32]. Additionally, in our study, *ITGB5* was also regulated by *LEF1*. These results suggest that *ITGB5* may have a significant function in the development of DN, via participating in cardiomyopathy pathways.

Additionally, a set of genes were markedly enriched in complement and coagulation cascades, such as *C1S* and *C1R*. These two genes both encode members of the human complement subcomponent C1, which is involved in immune response [33]. Migration of immune cells into the renal is a feature of early DN, and it is implicated in the development of this complication [34–37]. Previous studies also have found *C1S* and *C1R* to be differentially expressed in DN [38, 39]. In our study, *C1S* and *C1R* were regulated by *LEF1* and hsa-miR-33a. MiR-33a has been reported to be implicated in diabetes due to its regulation of insulin signaling and fatty acid metabolism [40, 41]. Therefore, *C1S* and *C1R* may be crucial in the development of DN.

However, this study has some limitations. For example, these predictions were not validated by experiments. The number of samples used for analysis is small. In further studies, more samples will be analyzed, and the predictions will be validated by experimental data.

## Conclusion

In conclusion, a total of 14 up-regulated genes and 430 down-regulated ones were identified. Some DEGs related to cytoskeleton organization (e.g. *MTSS1*, *ACTN4* and *CALD1*), cardiomyopathy (e.g. *ITGB5*) and immune response (e.g. *C1S* and *C1R*), as well as some regulators (e.g. *LEF1* and hsa-miR-33a) might play pivotal roles in the progression of DN. These findings may contribute to our better understanding of DN pathogenesis, and provide a theoretical basis for further experimental studies.

## Additional file

Additional file 1: The list of differentially expressed genes. (XLSX 30 kb)

## Abbreviations

ARVC: Arrhythmogenic right ventricular cardiomyopathy
BP: biological processes
CC: Cellular component
DEGs: Differentially expressed genes
DKD: Diabetic kidney disease
DN: Diabetic nephropathy
EMT: Epithelial to mesenchymal transition
GO: Gene Ontology
ICAM1: Intercellular adhesion molecule 1
LIMMA: Linear Models for Microarray Data
MF: Molecular function
mTOR: Mammalian target of rapamycin
RMA: Robust Multi-array Average
TFs: Transcription factors
WEGO: Web Gene Ontology Annotation Plot
